# Current evidence of synaptic dysfunction after stroke: Cellular and molecular mechanisms

**DOI:** 10.1111/cns.14744

**Published:** 2024-05-10

**Authors:** Chuan Li, Min Jiang, Zhi‐Ting Fang, Zhiying Chen, Li Li, Ziying Liu, Junmin Wang, Xiaoping Yin, Jian Wang, Moxin Wu

**Affiliations:** ^1^ Department of Medical Laboratory Affiliated Hospital of Jiujiang University Jiujiang Jiangxi China; ^2^ Jiujiang Clinical Precision Medicine Research Center Jiujiang Jiangxi China; ^3^ Department of Pathophysiology, Tongji Medical College Huazhong University of Science and Technology Wuhan Hubei China; ^4^ Department of Neurology Affiliated Hospital of Jiujiang University Jiujiang Jiangxi China; ^5^ Department of Intensive Care Unit The Affiliated Hospital of Jiujiang University Jiujiang Jiangxi China; ^6^ Department of Human Anatomy, School of Basic Medical Sciences Zhengzhou University Zhengzhou Henan China

**Keywords:** astrocytes, microglia, stroke, synapse pruning, synaptic dysfunction

## Abstract

**Background:**

Stroke is an acute cerebrovascular disease in which brain tissue is damaged due to sudden obstruction of blood flow to the brain or the rupture of blood vessels in the brain, which can prompt ischemic or hemorrhagic stroke. After stroke onset, ischemia, hypoxia, infiltration of blood components into the brain parenchyma, and lysed cell fragments, among other factors, invariably increase blood–brain barrier (BBB) permeability, the inflammatory response, and brain edema. These changes lead to neuronal cell death and synaptic dysfunction, the latter of which poses a significant challenge to stroke treatment.

**Results:**

Synaptic dysfunction occurs in various ways after stroke and includes the following: damage to neuronal structures, accumulation of pathologic proteins in the cell body, decreased fluidity and release of synaptic vesicles, disruption of mitochondrial transport in synapses, activation of synaptic phagocytosis by microglia/macrophages and astrocytes, and a reduction in synapse formation.

**Conclusions:**

This review summarizes the cellular and molecular mechanisms related to synapses and the protective effects of drugs or compounds and rehabilitation therapy on synapses in stroke according to recent research. Such an exploration will help to elucidate the relationship between stroke and synaptic damage and provide new insights into protecting synapses and restoring neurologic function.

## Graphical abstract

Stroke can cause synaptic loss and dysfunction, reducing communication between neurons. Stroke is known to cause the death of neuronal and endothelial cells, trigger microglia and astrocyte activation, and lead to synaptic dysfunction.

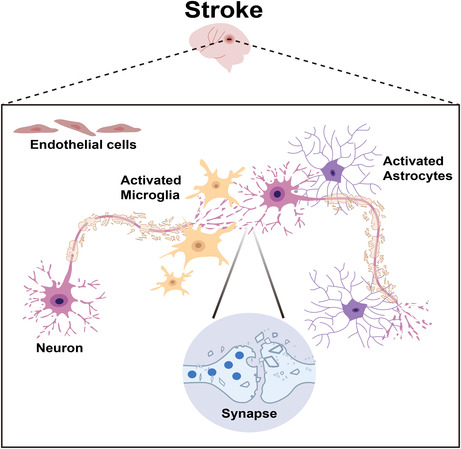

## 1 INTRODUCTION

Stroke, including both hemorrhagic and ischemic stroke, is an acute cerebrovascular disease in which brain tissue is damaged due to sudden obstruction of blood flow or rupture of blood vessels in the brain tissue. Globally, stroke is the second leading cause of death after cardiovascular disease, and in China, it is the leading cause of death and disability. Although epidemiological data from the past decade show no significant increase in stroke mortality, the global incidence and prevalence of stroke remain high.^1^ After stroke onset, injury factors, such as the infiltration of blood components into the brain parenchyma, lysed cellular debris, ischemia, and hypoxia, typically increase blood–brain barrier (BBB) permeability, the inflammatory response, and brain edema, which lead to neuronal cell death and synapse loss.^2^, ^3^ In stroke patients without intervention, 1.9 million neurons, 14 billion synapses, and 12 kilometers (7.5 miles) of myelinated nerve fibers are destroyed every minute, and 120 million neurons, 830 billion synapses, and 714 kilometers (447 miles) of myelinated nerve fibers are lost every hour,^4^ which contributes significantly to the high rate of mortality and disability in stroke patients.

The synapse, also called the neuronal junction, is the critical site of transmission of electric nerve impulses between two neurons or between a neuron and an effector cell (gland or muscle cell). Brain tissue accounts for only 2% of human body weight but consumes approximately one‐fifth of the body's energy, and most of that energy is used for synaptic signal transduction and neurotransmitter transport, which suggests that synapses are critical for organisms with brains.^5^ Synaptic dysfunction, which is associated with various neurological disorders, is an essential pathological hallmark of neurodegenerative disease and is recognized as the leading cause of cognitive impairment. After ischemic stroke, entire or specific regions of the brain become ischemic and hypoxic, which causes neuronal and synaptic damage and leads to neurological dysfunction. Two studies of human ischemic stroke patients have shown that synaptic density decreases within 1 month after stroke and further declines over time.^6^, ^7^ Yan et al.^8^ showed that in both middle cerebral artery occlusion (MCAO) and oxygen–glucose deprivation (OGD) mouse models of ischemia, synaptosome‐associated protein 29 (SNAP‐29) protein was expressed at low levels, and the volume of the presynaptic readily releasable pool (RRP) was reduced, which resulted in abnormal neurological function. Haghani et al.^9^ found that mild ischemia significantly increased basal synaptic transmission based on field potential recordings. However, a study by Li et al.^10^ showed that basal synaptic transmission decreased with 90 min of occlusion and 24 h of reperfusion. Similarly, after hemorrhagic stroke, hematoma compression or edema can adversely affect synapses. A study by Jang et al.^11^ revealed altered synapses in the brains of patients with hemorrhagic stroke. Using transmission electron microscopy (TEM), Li et al.^12^ found that the number and density of synapses in the striatum of mice in a collagenase‐induced intracerebral hemorrhage (ICH) model were abnormal. Synapse loss was observed on the first day and continued significantly through the third day, while synaptic density was further reduced on the 28th day. Wang et al.^13^ showed that synapses were also altered in ICH model rats, as a significant decrease in postsynaptic density protein 95 (PSD‐95) protein levels, which were observed in perihematomal cortical neurons at 6 h after ICH, reached a nadir at 12 h, and resulted in abnormal synaptic function. With advancements in medical technology, the mortality rate of stroke patients is gradually declining. Due to insufficient blood supply after stroke, the oxygen and glucose received by neurons are reduced; this interferes with the synthesis of adenosine 5′‐triphosphate (ATP) in mitochondria, which in turn causes synaptic damage.^14^, ^15^ However, the recovery period after treatment is long, and most patients experience poor recovery and have sequelae, including motor, sensory, speech, and cognitive dysfunction. These sequelae are often related to synaptic dysfunction caused by stroke. Therefore, protecting or reshaping synapses and restoring synaptic function during subsequent stroke treatment are critical strategies for intervention.

To the best of our knowledge, no review has comprehensively summarized the relationship between stroke and synaptic dysfunction. To better understand the mechanisms involved in synaptic dysfunction after stroke, we review the cellular and associated mechanisms that affect synapses as well as the drugs, compounds, and rehabilitation therapies that have demonstrated protective effects on synapses according to recent research.

## 2 CELLS INVOLVED IN REGULATING SYNAPTIC FUNCTION AFTER STROKE

**FIGURE 1:**
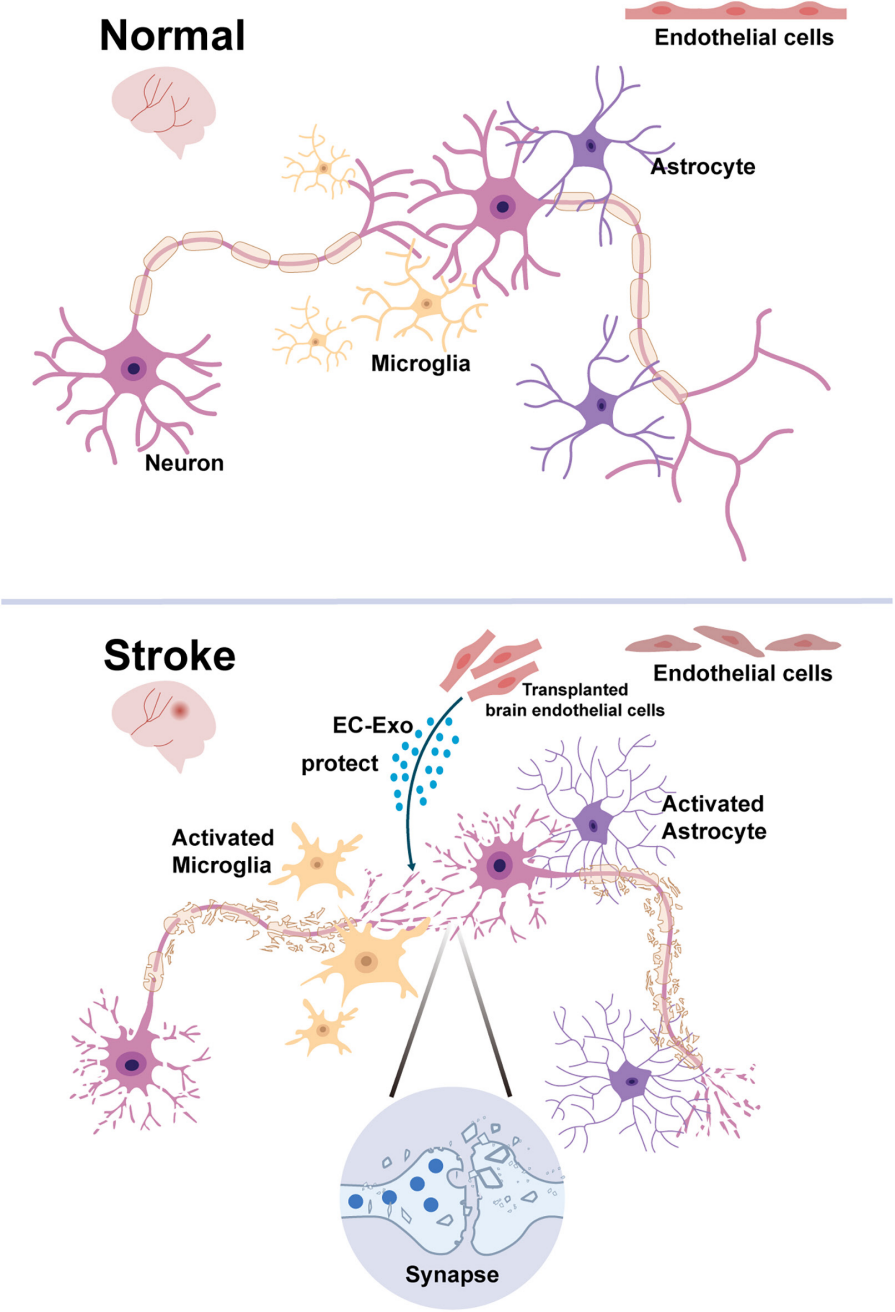
Different cells impact synaptic function after stroke. After stroke, neurons are destroyed and synapses are damaged, whereas microglia and astrocytes are activated. Microglia can cause damage to neurons and synapses, while astrocytes exert a protective effect, but excessive activation of astrocytes can aggravate synaptic damage. Exosomes produced by transplanted brain endothelial cells have certain protective effects on neurons and synapses.

Stroke causes brain tissue damage and neuronal cell death including neurons and endothelial cells, as well as the overactivation of microglia and astrocytes, which play important roles in maintaining the normal physiological functions of the brain. These cells respond differently after stroke and affect synapses, and thus, it is necessary to clarify their specific roles in synapses (Figure 1).

### 2.1 Neurons

As the basic signal processing unit of the human brain, neurons are electrically excitable cells that communicate with other cells via synapses, and their main function is to receive information and transmit it to other cells.^16^ After a stroke, neurons deprived of oxygen, energy, and metabolic substrates cease to function within seconds and show signs of structural damage after only 2 min, which manifests as degradation of the dendritic structure at the tip of the neuron.^17^ After stroke injury, astrocytes and nerve endings produce the high levels of glutamate within a few minutes, which is accompanied by reuptake impairment that ultimately leads to excessive glutamate accumulation.^18^ Neurons are sensitive to changes in glutamate levels.^19^ Activation of the ionotropic glutamate receptors N‐methyl‐D‐aspartate receptor (NMDAR) and α‐amino‐3‐hydroxy‐5‐methyl‐4‐isoxazolepropionic acid receptor (AMPAR) causes intracellular Ca^2+^ overload in neurons and triggers excitotoxicity,^20^, ^21^ which leads to mitochondrial dysfunction, nNOS/NOX2 activation, ROS/RNS generation, and ultimately damage to synaptic function.^22^, ^23^, ^24^ γ‐aminobutyric acid (GABA) is an important inhibitory neurotransmitter regulated by adenosine that maintains synaptic stability.^25^ Intermediate GABAergic neurons release GABA, which affects the conduction of excitatory signals by inhibiting the corresponding receptors of adjacent excitatory synapses to produce action potentials.^26^ In the early stage of ischemia/reperfusion (I/R), the loss of GABA‐mediated synaptic transmission in pyramidal neurons leads to increased cell excitability, promotes the opening of NMDAR channels, and subsequently disrupts the balance between excitatory and inhibitory neurotransmission, leading to neuronal death.^27^ In addition, reduced neurotrophic support can also lead to synaptic damage and neuronal death. The level of brain‐derived neurotrophic factor (BDNF) in the serum was shown to be significantly reduced in patients in the acute stroke stage, and the severity of stroke was negatively correlated with BDNF levels.^28^ During cerebral I/R, activation of the MAPK/ERK signaling pathway interacts with the PI3K/AKT signaling pathway and plays a key role in cell injury.^29^, ^30^

**FIGURE 2:**
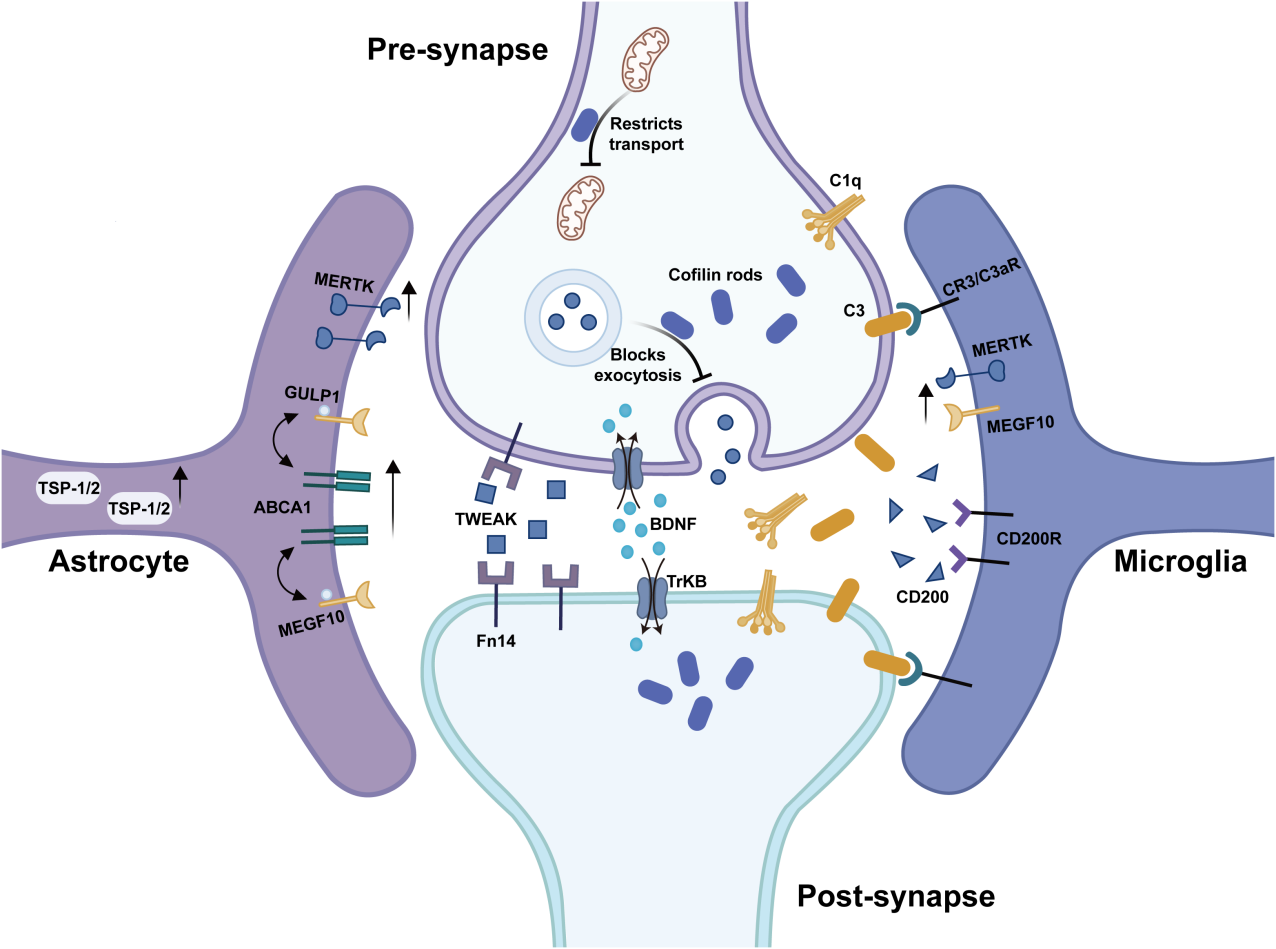
Synapse‐related signaling pathways in stroke. The formation of cofilin rods in neurons leads to the failure of mitochondrial transport in dendrites and affects synaptic function. The upregulation of TWEAK/Fn14 expression inhibits synaptic transmission and plasticity. Synapses are marked by increased levels of complement C1q and C3, which are recognized by microglia and then engulf the synapses. Activation of the CD200/CD200R pathway can improve the activation of microglia and the inflammatory environment, thereby promoting neurogenesis and functional recovery. The protein levels of MERTK and MEGF10 are increased, and astrocytes can upregulate ABCA1 and its pathway‐related molecules MEGF10 and GULP1 to transform into a phagocytic phenotype capable of engulfing synapses. An increase in TSP‐1/2 expression by astrocytes plays a key role in synaptic remodeling after stroke. Activation of the BDNF/TrkB signaling pathway also contributes to the recovery of synaptic function.

Presynaptic and postsynaptic injury caused by stroke is the early pathophysiologic basis of synaptic transmission disorders as well as neuronal cell death. In contrast, structural damage in neurons can lead to synaptic structure alterations, and pathological protein aggregation in neurons can also cause damage to synaptic structure and function. Cofilin is a small protein (19 kDa) that belongs to the actin‐depolymerizing factor/cofilin family. Cofilin rods, rod‐like pathological structures composed of cofilin and actin, have been shown to disrupt dendritic transport, leading to synapse loss and dysfunction.^31^ A large amount of cofilin rod accumulation may be found in neurons in the peri‐infarct region of rats used in MCAO and reperfusion (MCAO/R) models. Cofilin rods have also been observed in the dendrites of OGD‐treated neurons, where they lead to failure of mitochondrial transport in dendrites as well as damage to synaptic structure and function (Figure 2).^32^ SNAP‐29, an important protein involved in multiple membrane trafficking steps, was shown to be reduced in both the MCAO/R and OGD models. In one study, the mouse hippocampal CA1 region showed damage to the hippocampal‐mPFC (medial prefrontal cortex) loop and abnormal neurological function, and presynaptic excitatory transmission in primary cortical neurons was reduced after SNAP‐29 knockdown. Moreover, the size of the RRP at presynaptic sites decreased after SNAP‐29 knockdown.^8^ Overall, neurons provide the energy and material basis of synapses, and consequently, damage to neurons after stroke has a significant impact on synapses.

### 2.2 Microglia/macrophages

As resident immune cells of the central nervous system (CNS), microglia maintain homeostasis of the brain environment by monitoring the CNS.^33^ Once injury‐related signals are received, microglia are activated and migrate to the injury site.^34^, ^35^, ^36^, ^37^ In addition, microglia act as dynamic monitors of synapses, and abnormal transmission or dysfunctional synapses can trigger a microglial response.^38^

Histopathologic studies of human subacute‐phase stroke specimens have demonstrated extensive microglial activation surrounding the injury area.^39^ Microglial activation occurs within hours, and proinflammatory factors are released in response to synaptic degeneration or loss after stroke.^40^, ^41^ After stroke, various proinflammatory factors are released by microglia, such as TNF‐α, IL‐1β, IL‐6, IFN‐γ, chemokines, nitric oxide (NO), reactive oxygen species (ROS), and matrix metalloproteinase‐9 (MMP‐9), which further exacerbate the neuroinflammatory response and damage in the brain.^42^, ^43^, ^44^ Microglia/macrophages mediate synaptic elimination via the classical complement pathway in developing or diseased brains.^45^, ^46^ Generally, microglia/macrophage‐mediated phagocytosis is necessary to clear synaptic debris and is beneficial for brain recovery. Dead neurons and synaptic debris labeling have been observed in microglia/macrophages in stroke brains.^47^ A two‐photon imaging study using in vivo fluorescent labeling of neurons and microglia revealed that the activity‐dependent connection between microglia and synapses was significantly prolonged for approximately 1 h after cerebral ischemia. In contrast, the connection in the intact brain was maintained for approximately 5 min, which suggests that microglia can regulate long‐term potentiation (LTP).^34^ Microglia play a key role in the reconstruction of neural circuits after cerebral ischemic injury. However, microglia/macrophage phagocytosis damages surviving neurons and viable synapses in patients with Alzheimer's disease (AD) and stroke,^48^, ^49^ and inhibition of microglial activation can improve neuroinflammation and neuronal apoptosis, which prevents synaptic pruning, attenuates brain damage, and improves neurobehavioral outcomes.^50^, ^51^ Microglia/macrophages phagocytose viable neurons via milk fat globule‐EGF factor VIII (MFG‐E8) and MER proto‐oncogene tyrosine kinase (MERTK) during the acute phase of ischemic stroke, and inhibition of this process prevents delayed loss of functional neurons and death.^49^ Yang et al.^52^ further demonstrated that microglia can cause synaptic loss through phagocytosis. Using TEM and immunofluorescence staining, microglia were found to mediate synaptic phagocytosis in mice with ischemic and hemorrhagic stroke. They found that the cytoplasm of microglia/macrophages contained synaptic elements, including presynaptic (synaptophysin, SYP) and postsynaptic (Homer‐1) proteins, and that these cells engulfed both inhibitory and excitatory synapses in an MCAO model of ischemic stroke. Moreover, the width, length, and number of dendritic spines were significantly reduced, and the phagocytic activity of microglia/macrophages reached a peak on day 3 and then gradually decreased from day 7 to day 14. In a collagenase‐induced ICH mouse model, the phagocytic behavior of microglia/macrophages was similar to that in ischemic stroke. In that study, inhibition of microglia/macrophage phagocytosis significantly increased the synaptic density in the peripheral zone after injury, which led to improvements in motor and cognitive functions in the mice. Another study using a mouse model of ischemic stroke revealed that microglia mediated synaptic pruning through the complement system, which led to reduced synaptic density and cognitive decline.^53^ In summary, microglia exhibit similar phagocytic activity of synapses after ischemic and hemorrhagic stroke and play a key role in synaptic dysfunction. Blocking or inhibiting microglial phagocytosis can rescue synaptic loss, reduce brain damage, and improve motor and cognitive function.

### 2.3 Astrocytes

Astrocytes are glial cells that participate in several physiological and pathological processes in the CNS. Under normal physiological conditions, astrocytes are a key component of the BBB and participate in and maintain the stability of the CNS microenvironment.^54^ In addition, astrocytes secrete cytokines that regulate the survival and differentiation of neurons and the formation and elimination of synapses.^55^, ^56^

During postnatal development, astrocytes eliminate synapses by phagocytosis through the EGF‐like domain 10 (MEGF10) and MERTK receptors and actively contribute to activity‐dependent synaptic pruning and developmental refinement of circuits.^57^ Moreover, contrary to the previous notion that microglia are the sole mediators of synapse elimination, astrocytes have been shown to play an important role in synaptic pruning during brain development.^46^, ^58^, ^59^ Each astrocyte occupies a separate and nonoverlapping region in the brain. Astrocytes interact with synapses to form a precise and complex network. However, stroke disrupts this relatively independent relationship, as dead neurons and synaptic fragments are observed in astrocytes after stroke.^60^

Astrocytes have been shown to block damage caused by stroke and other diseases of the CNS, including suppression of synaptic damage and amelioration of synaptic dysfunction.^61^ Astrocytes play a critical role in regulating synaptic remodeling after stroke. In a mouse model of focal cerebral ischemia, thrombospondin 1 and 2 (TSP‐1/2) expression was significantly increased and colocalized mostly to astrocytes, whereas inhibition of TSP‐1/2 expression reduced synaptic density and defective axon outgrowth (Figure 2),^62^ which suggests that TSP‐1/2 expression in reactive astrocytes exerts a protective effect on synapses.

Mounting evidence indicates that astrocytes undergo significant changes in morphology, gene expression, and cell proliferation after stroke, at which point these cells are termed reactive astrocytes (RAs).^63^ Currently, the functions of RAs include scar formation, neurotrophic factor secretion, BBB injury and repair, and inhibition of synapse formation. However, the generation and mechanisms of Ras are still unclear.^64^ Ras are considered neurotoxic, and their presence after stroke is correlated with poor neurological prognosis. Recent studies have shown that newly formed synapses can be engulfed by Ras, thereby inhibiting the recovery of neurological function after stroke. In ischemic stroke models, astrocytes convert to a phagocytic phenotype by upregulating ATP‐binding cassette transporter A1 (ABCA1) and its pathway‐related molecules MEGF10 and engulfment adapter phosphotyrosine‐binding domain containing 1 (GULP1), which can engulf presynaptic and postsynaptic components (Figure 2). Disruption of ABCA1 in RAs can reduce astrocyte phagocytosis and brain damage and improve neurobehavioral outcomes.^60^ The cytoplasm of astrocytes contains synaptic elements, including the synaptic proteins SYP and Homer‐1, and in an MCAO mouse model, astrocytes were shown to engulf both inhibitory and excitatory synapses. In that study, the phagocytic capacity of astrocytes gradually increased from day 1 to day 14. However, in a collagenase‐induced ICH mouse model, only a few astrocytes exhibited phagocytic activity, with an obvious decrease in the phagocytic effect on both inhibitory and excitatory synapses. In addition, a comparative transcriptomics analysis of posthemorrhagic and ischemic stroke revealed significant differences in phagocytosis‐associated gene expression and biological processes in astrocytes.^52^ The latest visualization technology allows the direct observation of the ultrastructures of astrocytes and allows imaging of the tiny projections of astrocytes and some of their synaptic proteins. This method can be used to visualize the structure of astrocytes in the range of tens of nanometers and has good potential for observing the interaction between astrocytes and synapses after stroke.^65^

Overall, astrocytes act as a double‐edged sword on synapses after stroke. On the one hand, astrocytes have a protective effect on synapses and repair synaptic function after stroke. On the other hand, overactivated astrocytes can phagocytose labeled synapses and newly formed synapses, further exacerbating synapse loss.

### 2.4 Endothelial cells

Communication and signal transduction between cells in the brain are the basis for functional homeostasis of the CNS. Endothelial cells, which are distributed throughout the vascular network, are the “first responder cells” to hypoxic stress and are regulated mainly by paracrine signals in brain tissue.^66^ However, due to the complexity of neurovascular interactions, the understanding of endothelial cell regulation after stroke is still in its infancy.

The impairment of cerebral microvascular endothelial cells is an early manifestation of BBB injury caused by cerebral I/R injury, which increases the permeability of the BBB, promotes the occurrence of cerebral edema, and is detrimental to the prognosis of stroke patients.^67^ Endothelial progenitor cells (EPCs) are immature endothelial cells that proliferate and differentiate into mature endothelial cells. Ma et al.^68^ revealed that EPC transplantation reduces the expression of the astrocyte‐derived C3/C3aR pathway inflammatory response in the brain after ischemic stroke, which contributes to the recovery of neurological function. EPC transplantation increases CR3‐mediated microglia/macrophage phagocytosis and SYP and PSD‐95 expression, subsequently attenuating synaptic loss under oxygen–glucose deprivation conditions and in adult male mice with transient MCAO.^69^ In addition, exosomes derived from brain endothelial cells (EC‐Exos) protect neurons from hypoxic injury. Recent studies have shown that EC‐Exos inhibit neuronal apoptosis and increase synapse length after oxygen–glucose deprivation and reperfusion (OGD/R). EC‐Exos not only improve neuromotor behavior and increase regional cerebral blood flow (rCBF) but also promote the expression of synaptic regulatory proteins and inhibit apoptosis in MCAO/R‐injured mouse brains.^70^ In conclusion, research on how endothelial cells affect poststroke synapses is still relatively insufficient. However, studies have shown the positive effects of EPC transplantation and EC‐Exos on synapses, which may serve as a new approach for the treatment of synaptic dysfunction after stroke.

## 3 SYNAPSE‐RELATED SIGNALING PATHWAYS IN STROKE

### 3.1 Complement system

The complement system, also known as the complement cascade, is an important part of the innate immune system. The complement system not only participates in various activities of the immune system but also plays an important role in the brain injury process of neurological diseases such as Alzheimer's disease, traumatic brain injury (TBI), and ischemic and hemorrhagic stroke.^2^, ^71^

The classical complement cascade acts as a “marker” for synaptic pruning by microglia in the normal brain. The initial complement protein C1q and the central complement protein C3 are located at synapses where they mediate synaptic elimination by phagocytic microglia.^72^ Several studies have shown that excessive complement accumulation can lead to abnormal activation of the synaptic pruning function of microglia, which results in the engulfment of a large number of synapses and eventually leads to synaptic loss and neuronal cell death.^45^, ^73^, ^74^ During brain injury and aging, the complement cascade is activated, which leads to an increase in complement C1q and C3 levels around synapses; this mediates synaptic pruning by binding to CR3 or C3aR receptors on the surface of microglia and leads to synaptic loss and neuronal cell death (Figure 2).^45^, ^74^, ^75^, ^76^ Data from human stroke patients have revealed significant deposition of complement C1q and C3d components in ischemic brain tissue and that the levels of the activated complement proteins C5a and C3a in serum are correlated with the severity of pathological and clinical outcomes.^77^, ^78^, ^79^ Microglia achieve synapse elimination through the complement system, and the inhibition of complement system activation blocks synapse loss.^80^ Complement activation and opsonization at hippocampal synapses directly lead to microglia‐dependent synaptic phagocytosis and a decrease in synaptic density after stroke. Inhibition of complement activation or microglial phagocytosis can reverse synaptic loss, attenuate brain injury, and improve neurobehavioral outcomes in MCAO and collagenase‐induced ICH mouse models.^52^, ^53^ Using a murine microthrombus stroke model, Alawieh et al.^53^ reported that B4Crry, an inhibitor of complement C3, can limit perilesional complement deposition, reduce microgliosis and synapse uptake, and improve cognitive outcomes. Wu et al.^81^ reported that plasma C3 levels are elevated in patients with ICH and are closely related to hemorrhagic severity and clinical outcomes. Normobaric hyperoxia therapy exerts neuroprotective effects by reducing C3‐mediated synaptic pruning in ICH patients and in a collagenase‐induced mouse model. In addition, plasma C4 levels are significantly elevated and are associated with clinical outcomes in patients with hemorrhagic stroke.^82^ More importantly, C4 is critical for synaptic pruning, and variations in C4 induce excessive neuronal complement deposition and contribute to increased microglial synapse uptake in cocultured human iPSC‐derived neurons and microglia.^83^ Two studies involving mouse models also revealed that overexpression of the schizophrenia‐associated gene C4 promotes excessive synaptic loss and behavioral changes through microglial synaptic engulfment.^84^, ^85^ Thus, C4 plays an important role in secondary brain injury, but further research is needed to determine whether microglia induce secondary brain injury through C4‐mediated synaptic pruning.

Inhibition of complement system activation helps to reduce the phagocytic effect of synapses by microglia, attenuate brain injury, promote early recovery of neurological function after brain injury, and improve neurobehavioral outcomes. However, the specific complement components involved in microglia‐mediated synaptic pruning after stroke have not yet been fully elucidated.

### 3.2 The MEGF10/MERTK pathway

MEGF10 is an ortholog of Drosophila Draper and the *C. elegans* protein CED‐1, which help to mediate axon pruning by Drosophila glial cells and phagocytosis of apoptotic cells by worms, respectively.^86^, ^87^ MEGF10 has also been shown to mediate phagocytosis through the involvement of other proteins, such as GULP1 and ABCA1, but little is known about the function of GULP1 in this pathway.^88^, ^89^, ^90^ MERTK, a member of the MER/AXL/TYRO3 receptor kinase family, mediates the shedding of the photoreceptor outer segment by retinal pigment epithelial cells.^91^, ^92^ MERTK cooperates with the integrin pathway to regulate the CRKII/DOCK180/Rac1 module and control the rearrangement of the actin cytoskeleton during phagocytosis.^93^, ^94^

Both MEGF10 and MERTK exert phagocytic effects by recognizing the “eat‐me” signal. Chung et al.^57^ showed that astrocytes engulf synapses through the MEGF10 and MERTK pathways and contribute to activity‐dependent synaptic elimination, thereby mediating the development of CNS neural circuits, and that astrocytes continue to engulf synapses in the adult CNS. A recent study revealed that MEGF10 and MERTK were expressed on the membranes of microglia/macrophages and astrocytes in mice with ischemic and hemorrhagic stroke (Figure 2) and that MEGF10 and MERTK levels were significantly increased 14 days after stroke. Conditional knockout of MEGF10 or MERTK in microglia/macrophages attenuated the phagocytosis of synapses, which was shown to be helpful for improving dendritic spine structure and neurological dysfunction in an ischemic and hemorrhagic stroke mouse model. However, conditional knockout of MEGF10 or MERTK in astrocytes inhibited the phagocytosis of synapses, improved dendritic spine structure, reduced brain injury and improved neurobehavior in ischemic stroke but had no significant effect on hemorrhagic stroke.^52^ This finding suggests that the MEGF10 and MERTK pathways play key roles in mediating synaptic pruning and provides new strategies for protecting synapses after stroke.

### 3.3 The TWEAK/Fn14 pathway

The tumor necrosis factor‐like weak inducer of apoptosis (TWEAK) protein was originally discovered as a cytokine produced by macrophages that signals through the damage‐induced transmembrane receptor fibroblast growth factor‐inducible 14 (Fn14).^95^, ^96^ The function of the TWEAK/Fn14 signaling pathway has been defined as a driving factor for tissue remodeling in multiple organ systems in the context of injury and disease.^97^

The TWEAK/Fn14 signaling pathway was recently shown to be required for synapse maturation during experience‐dependent visual development.^98^ The upregulation of Fn14 in thalamocortical excitatory neurons induced by light exposure and the corresponding increase of TWEAK in microglia collectively orchestrate the elimination of weaker synapses and reinforce the strength of the remaining synapses in the dorsal lateral geniculate nucleus (dLGN).^99^ In addition, it has been shown that the TWEAK/Fn14 pathway induces neuronal cell death in the CNS, and significant upregulation of TWEAK and Fn14 was reported in the CNS and cerebrospinal fluid in patients with ischemic stroke (Figure 2).^100^, ^101^, ^102^ In the MCAO plus hypoxia mouse model of ischemic stroke, a combination of electrophysiological and phosphorylated proteomic approaches revealed that TWEAK acutely inhibits basal synaptic transmission and plasticity through neuronal Fn14 and affects the phosphorylation of pre‐ and postsynaptic proteins in hippocampal brain slices from adult mice. However, the inhibition of TWEAK/Fn14 signaling‐related synaptic function may be augmented in an ischemic stroke model.^103^ These studies suggest that the TWEAK/Fn14 signaling pathway could be a potential target for the treatment of synaptic dysfunction after stroke.

### 3.4 The CD200/CD200R pathway

As a member of the immunoglobulin superfamily, CD200 is widely expressed in neurons, astrocytes, and oligodendrocytes, while its receptor (CD200R) is expressed in myeloid cells and microglia in rodents and is highly expressed in neurons in humans.^104^ The CD200/CD200R pathway plays a critical regulatory role in neural recovery under various pathological brain conditions because of its unique expression profile. It has been shown that the CD200/CD200R signaling pathway is involved in regulating synaptic plasticity, and dysfunction of this pathway leads to synaptic deficits in aging and AD.^105^, ^106^ Suppressing the CD200/CD200R signaling pathway through genetic approaches impairs LTP,^107^ whereas activating CD200/CD200R signaling through pharmacological methods significantly enhances synaptic plasticity by decreasing neuroinflammation in AD and aged mice.^105^, ^106^, ^108^ In a transient MCAO rat model, the CD200/CD200R signaling pathway was shown to be activated, and inhibition of microglial activation and the release of inflammatory factors by CD200Fc (a CD200R agonist) resulted in the preservation of synapse‐associated proteins and dendritic spines and was accompanied by the restoration of sensorimotor function (Figure 2).^104^ In the same model, CD200 and CD200R levels in the ipsilateral hippocampus and cortex were elevated after treadmill exercise, which improved the inflammatory environment and promoted neurogenesis and functional recovery after stroke.^109^ Thus, the CD200/CD200R signaling pathway plays an important role in neurological recovery by regulating synaptic plasticity in stroke, which is worthy of further study.

## 4 DRUGS AND COMPOUNDS TO IMPROVE SYNAPTIC FUNCTION AFTER STROKE

### 4.1 Folic acid

Folic acid, a water‐soluble B vitamin, has been shown to reduce the risk of first stroke among adults with hypertension and to improve associated poor outcomes in randomized clinical trials.^121^ Liang et al.^113^ reported that folic acid‐supplemented diets reduced neuronal cell death and p‐CAMII levels, increased synapse numbers and the expression of the presynaptic proteins GAP‐43, SYN, and SNAP25 and the postsynaptic protein PSD‐95, and improved cognitive performance in a rat model of ischemic MCAO/R. After folic acid treatment, similar changes in synaptic function were also observed in OGD/R‐treated neurons in an in vitro model. Another study showed that folic acid improves I/R‐induced synaptic damage by inhibiting the excessive activation of NMDARs (Table 1).

**TABLE 1:** Drugs or compounds that can reduce synaptic damage after stroke.

Treatment	Target/pathway	Mechanism	Results	References
B4Crry	Complement 3	Reduces perilesional complement deposition and microglial proliferation as well as microglial uptake into synapses	Improves the safety and efficacy of thrombolytic therapy, inhibits complement‐dependent neurodegeneration, and improves chronic cognitive outcomes, manifested as low neurological deficit scores	[53]
Minocycline	mTOR signaling	Inhibits mTOR signaling, enhances the autophagy process, and promotes the expression of presynaptic and postsynaptic proteins (SYN and PSD‐95)	Prevents cognitive decline in rats that undergo MCAO	[110]
GJ‐4	JAK2/STAT1 pathway	Upregulates the expression of SYP and PSD‐95 and downregulates the expression of NMDAR1	Reduces brain damage, improves neurological dysfunction, and alleviates learning and memory impairment in stroke rats	[111]
Neural Fuyuan Formula	BDNF/TrKB signaling pathway	Upregulates the expression of BDNF signaling protein and synapse‐related proteins and increases the expression of SYN‐I in neurons	Reduces synaptic damage, promotes recovery of neurological function, and improves poststroke depression in rats	[112]
Folic acid	NMDA receptors	Inhibits the excessive activation of NMDA receptors, reduces the expression of p‐CAMKII, and upregulates the expression of synapse‐related proteins (SNA‐25, SYN, GAP‐43, PSD‐95)	Reduces neuronal cell death, increases the number of synapses, and ameliorates learning and memory deficits induced by brain ischemia	[113]
Oleanolic Acid	/	Inhibits astrocyte proliferation and microglia activation, promotes the expression of synapse‐related proteins, and increases the number of DCX + cells in the hippocampus	Reduces brain loss, promotes the recovery of neurological functions, and improves motor functions and learning and memory abilities	[114]
Cocaine‐ and amphetamine‐regulated transcript (CART)	CREB	Upregulates the expression of p‐CREB and Arc	Alleviates ischemia‐induced neuronal synaptic damage and increases presynaptic vesicle number and postsynaptic density	[115]
Baishaoluoshi Decoction	BDNF/TrKB‐KCC2 pathway	Upregulates the expression of BDNF, TrKB, and KCC2 in the periphery of infarct and brainstem	Reduces neuronal and synaptic damage and improves the spasticity of hemiplegic limbs in poststroke spasticity model rats	[116]
Xiaoxuming Decoction	/	Upregulates the expression of SYP and PSD‐25	Reduces the formation of cerebral infarct area, improves synaptic plasticity in the ischemic penumbra during acute cerebral ischemia–reperfusion, and significantly reduces Longa score	[117]
Huangqi Guizhi Wuwu decoction	Sirt1/NF‐κB/NLRP3 pathway	Upregulates the expression of Sirt1 and inhibits the expression of p‐NF‐κB, NLRP3, ASC, and cleaved caspase‐1. Inhibits M1 polarization of microglia, promotes M2 polarization of microglia, and increases the levels of synaptic marker proteins (PSD‐95, SYP‐I)	Reduces the cerebral infarct area, increases the density of dendritic spines, and improves the neurological dysfunction of rats with stroke, manifested as high mNSS scores	[118]
Cerebrolysin	/	Increases GAP‐43, PSD‐95, and SYP levels in the lesion area	Improves synaptic plasticity and cognitive function after mPFC ischemia, alleviates mPFC ischemia‐induced episodic and spatial memory impairment	[119]
Alpha‐Asarone	CaMKII‐dependent pathways	Reduces calcium overload and CaMKII phosphorylation in the acute phase, inhibits mitochondrial‐involved apoptosis, increases the number of synapses in the ipsilateral hippocampus during recovery, and enhances synaptic plasticity	Reduces the mortality rate of rats with subarachnoid hemorrhage and the seizure rate of rats with epilepsy within 24 h, prevents neuronal damage after subarachnoid hemorrhage, improves the neurological dysfunction and ameliorates Garcia and beam balance scores of rats	[120]

### 4.2 Oleanolic acid

Oleanolic acid (OA), a natural pentacyclic triterpenoid compound, is a bioactive component of ginseng that can cross the BBB.^122^ Lin et al.^123^ reported that OA administration could protect neurons in an OGD/R neuron model and attenuate ischemic injury by reducing oxidative stress in an MCAO rat model. Over the short term, OA alleviated the cerebral infarct area, neurological symptoms, and expression of MMP‐9 and occludin and blocked malonaldehyde generation at 24 h in an MCAO mouse model. Over the long term, daily injection of OA significantly reduced brain loss, inhibited astrocyte proliferation and microglia activation, and promoted the expression of synaptic‐related proteins and synaptic connections, thereby promoting the recovery of neurological function and improving learning and memory in the hippocampus.^114^ OA is a promising neuroprotective drug with potential value in the treatment of synaptic dysfunction after stroke. However, further research is needed to define how OA inhibits the proliferation and activation of glial cells, thereby protecting synapses.

### 4.3 Agrin

As a proteoglycan, agrin aggregates on acetylcholine receptors (AChRs) at neuromuscular junctions and participates in synaptogenesis during CNS development.^124^ Synaptogenesis is an important and beneficial factor in the recovery of behavioral function after stroke. Zhang et al.^125^ demonstrated that in an MCAO ischemic rat model, exercise promoted behavioral functional recovery, which was accompanied by upregulated expression of agrin and increased synaptic density. In vitro studies of an OGD‐induced ischemic neuron model revealed that agrin induces synaptogenesis and that a cAMP response element binding protein (CREB) inhibitor downregulates the expression of agrin and impedes synaptogenesis. However, the underlying mechanisms of agrin‐induced neuroplasticity and synaptogenesis are unclear and require further investigation.

### 4.4 Cocaine‐ and amphetamine‐regulated transcripts (CARTs)

CART is a neuropeptide that exerts neuroprotective effects in animal models of cerebral I/R injury and in OGD‐cultured neurons. Wang et al.^126^ showed that CART treatment reduces neuronal cell apoptosis induced by OGD injury and repairs OGD‐injured cortical neurons by enhancing the expression of growth‐associated protein 43 (GAP‐43), which promotes neurite outgrowth through a pleiotrophin‐dependent pathway. Zhang et al.^115^ reported that CART treatment increases the survival rate of neurons, significantly attenuates synaptic damage, and increases the expression of SYP in an OGD neuron model. Additionally, after CART treatment, the postsynaptic density increases, as does the number of presynaptic vesicles. Mechanistically, CART treatment increased the expression of Arc mRNA in a CREB‐dependent manner. CART therapy has a protective effect on the synaptic structure of neurons after ischemic brain injury and has potential value in the treatment of poststroke synaptic dysfunction.

### 4.5 Other agents

In several recent studies, Salvia miltiorrhiza (SM) was shown to alleviate synaptic deficits and neuronal loss in a transient MCAO mouse model.^127^ Rhynchophylline attenuates sensorimotor deficits, alleviates hippocampus‐dependent spatial memory injury, and reduces infarct volume in mice that undergo MCAO. In addition, rhynchophylline administration ameliorates the loss of synaptophysin I and improves dendritic complexity, dendritic spine density, and synaptic plasticity in mice that undergo MCAO.^128^ Xiaoxuming decoction may improve the synaptic plasticity of the ischemic penumbra during acute cerebral I/R injury by upregulating the expression of SYP and PSD‐95.^117^ Both Neural Fuyuan Formula and Baishaoluoshi Decoction can activate the BDNF/TrKB signaling pathway, which effectively reduces damage to neurons and synapses after stroke and promotes the recovery of neurological function (Figure 2).^112^, ^116^ In addition, Chinese herbal medicine also has a positive effect on poststroke synaptic dysfunction.^129^, ^130^ However, further clinical trials are needed to test the clinical application of these drugs.

## 5 REHABILITATION THERAPY TO IMPROVE SYNAPTIC DYSFUNCTION

The recovery period after stroke is long, and most patients do not experience satisfactory recovery and have sequelae. Intervention during the recovery period through various methods may promote patient recovery. Acupuncture has been shown to alter synaptic structure and promote synaptic plasticity by upregulating SYP and increasing postsynaptic densities in rodent models of stroke.^131^ Repetitive transcranial magnetic stimulation (rTMS) also has positive effects in animal models of ischemic stroke. rTMS can promote synaptic plasticity and thus induce neurological recovery by increasing the expression of NMDAR, AMPAR, and BDNF.^132^ Recent research has shown that stroke patients exhibit significant improvements in movement disorders, motor function, and quality of life after rTMS,^133^, ^134^ but whether this approach improves patient outcomes by affecting synaptic function requires further research. Increasing numbers of studies have shown that proper exercise can aid recovery after stroke. Li et al.^135^ showed that exercise promotes the expression of synaptic plasticity‐related proteins by regulating exosomal content, which increases the number of synapses in the rat brain and thus promotes the recovery of motor function in an MCAO rat model. Moreover, subsequent experiments by Li et al.^136^ demonstrated that exercise intervention regulates synaptic plasticity via inhibition of overactivated microglia via exosomes. Chen et al.^137^ showed that, in an MCAO mouse model, exercise promotes synaptic proliferation and ultimately leads to nerve regeneration by converting astrocytes into neuroprotective reactive astrocytes. All the above methods affect poststroke synapses, but further research is needed before they are applied in the clinic.

## 6 CONCLUSIONS

Although synapses play a crucial role in brain function, their role has been largely ignored in previous studies on stroke pathogenesis and rehabilitation mechanisms. To date, it has been reported in clinical practice that neuron‐centered treatment strategies lack effectiveness in reducing infarction or improving functional recovery. Therefore, a comprehensive understanding of the effects of stroke on synaptic function is necessary to develop more effective therapeutic strategies.

In this review, we focus on the cells and mechanisms that influence synaptic function after stroke. Synapses are considered vulnerable sites on neurons, and in many brain diseases, synaptic damage precedes the beginning of neuronal cell death.^5^ After stroke, neuronal structure is damaged, and pathological aggregation of intracellular proteins causes damage to synaptic structure and function. Typically, phagocytosis mediated by microglia/macrophages is thought to be necessary for the removal of synaptic debris and to favor brain recovery. However, microglial phagocytosis also has a detrimental effect on synapses, and the release of proinflammatory factors can respond to synaptic degeneration or loss via impairment of neural function in the brain. Microglia mediate synaptic phagocytosis after stroke through the complement system, but the specific complement molecules that mediate synaptic pruning by microglia have not been fully elucidated and require further study. Astrocytes secrete factors that regulate synapse formation, function, and elimination and can utilize the MEGF10/MERTK receptor phagocytosis pathway to mediate synapse elimination in the brain. Inhibition of this pathway inhibits astrocyte phagocytosis, which in turn attenuates brain damage and improves neurobehavior. Studies have shown that transplantation of EPCs and exosomes derived from brain endothelial cells is beneficial for synapses after stroke, but the mechanisms involved and the specific components of the exosomes require further study. In addition, we also mentioned the substances and methods that have been shown to have a positive effect on synapses in recent stroke studies. Some of these substances or methods can effectively improve poststroke synaptic dysfunction in animal experiments, and further clinical trials can be performed to identify new strategies for the treatment of poststroke synaptic dysfunction. Generally, synapses are severely damaged after stroke, and it is necessary to clarify the relationship between synapses and surrounding cells to explore strategies to improve synaptic dysfunction and the sequelae of stroke.

## AUTHOR CONTRIBUTIONS

The initial idea for this review was conceived by M.X.W., J.W., X.P.Y., and C.L., and the manuscript was written and revised by C.L., M.X.W., M.J., and Z.T.F. In addition, Z.Y.C., L.L., Z.Y.L., and J.M.W. contributed to the preparation of the illustrations; J.W. and M.J. edited the manuscript, and C.L., J.W., and M.X.W. read, reviewed, and approved the final manuscript. The final version of the manuscript was approved by all the authors.

## FUNDING INFORMATION

This study was supported partially by the National Natural Science Foundation of China (82260209 and 81960221 to XPY; 82371339 to JW); the Jiangxi Provincial Natural Science Foundation of China (20232BAB206046 to MXW); the Science and Technology Project Founded by the Education Department of Jiangxi Province (GJJ201834 to MXW); and the Jiangxi Provincial Health Commission Science and Technology Plan project (202212021 to MXW).

## CONFLICT OF INTEREST STATEMENT

Jian Wang is an Editorial Board member of CNS Neuroscience and Therapeutics and a coauthor of this article. To minimize bias, he was excluded from all editorial decision‐making related to the acceptance of this article for publication. The other authors declare that they have no conflicts of interest.

## CONSENT FOR PUBLICATION

Not applicable.

## Data Availability

Data sharing not applicable to this article as no datasets were generated or analysed during the current study.

## References

[1] Wu S , Wu B , Liu M , et al. Stroke in China: advances and challenges in epidemiology, prevention, and management. Lancet Neurol. 2019;18(4):394‐405. 30878104 10.1016/S1474-4422(18)30500-3

[2] Iadecola C , Buckwalter MS , Anrather J . Immune responses to stroke: mechanisms, modulation, and therapeutic potential. J Clin Invest. 2020;130(6):2777‐2788. 32391806 10.1172/JCI135530 PMC7260029

[3] Ren H , Han R , Chen X , et al. Potential therapeutic targets for intracerebral hemorrhage‐associated inflammation: an update. J Cereb Blood Flow Metab. 2020;40(9):1752‐1768. 32423330 10.1177/0271678X20923551 PMC7446569

[4] Saver JL . Time is brain–quantified. Stroke. 2006;37(1):263‐266. 16339467 10.1161/01.STR.0000196957.55928.ab

[5] Fedorovich SV , Waseem TV . Metabolic regulation of synaptic activity. Rev Neurosci. 2018;29(8):825‐835. 29768250 10.1515/revneuro-2017-0090

[6] Michiels L , Thijs L , Mertens N , et al. Longitudinal synaptic density PET with (11) C‐UCB‐J 6 months after ischemic stroke. Ann Neurol. 2023;93(5):911‐921. 36585914 10.1002/ana.26593

[7] Michiels L , Mertens N , Thijs L , et al. Changes in synaptic density in the subacute phase after ischemic stroke: a (11)C‐UCB‐J PET/MR study. J Cereb Blood Flow Metab. 2022;42(2):303‐314. 34550834 10.1177/0271678X211047759 PMC9122519

[8] Yan W , Fan J , Zhang X , et al. Decreased neuronal synaptosome associated protein 29 contributes to poststroke cognitive impairment by disrupting presynaptic maintenance. Theranostics. 2021;11(10):4616‐4636. 33754017 10.7150/thno.54210 PMC7978312

[9] Haghani M , Keshavarz S , Nazari M , Rafati A . Electrophysiology of cerebral ischemia and reperfusion: first evidence for the role of synapse in ischemic tolerance. Synapse. 2016;70(9):351‐360. 27124112 10.1002/syn.21910

[10] Li Z , Pang L , Fang F , et al. Resveratrol attenuates brain damage in a rat model of focal cerebral ischemia via up‐regulation of hippocampal Bcl‐2. Brain Res. 2012;1450:116‐124. 22410291 10.1016/j.brainres.2012.02.019

[11] Jang SH , Chang CH , Kim SH , Jung YJ , Hong JH . Thalamic reorganization in chronic patients with intracerebral hemorrhage: a retrospective cross‐sectional study. Medicine (Baltimore). 2015;94(34):e1391. 26313781 10.1097/MD.0000000000001391 PMC4602938

[12] Li Q , Weiland A , Chen X , et al. Ultrastructural characteristics of neuronal death and white matter injury in mouse brain tissues after intracerebral hemorrhage: coexistence of Ferroptosis, autophagy, and necrosis. Front Neurol. 2018;9:581. 30065697 10.3389/fneur.2018.00581 PMC6056664

[13] Wang Z , Chen Z , Yang J , et al. Treatment of secondary brain injury by perturbing postsynaptic density protein‐95‐NMDA receptor interaction after intracerebral hemorrhage in rats. J Cereb Blood Flow Metab. 2019;39(8):1588‐1601. 29513122 10.1177/0271678X18762637 PMC6681427

[14] Tian H , Chen X , Liao J , et al. Mitochondrial quality control in stroke: from the mechanisms to therapeutic potentials. J Cell Mol Med. 2022;26(4):1000‐1012. 35040556 10.1111/jcmm.17189 PMC8831937

[15] An H , Zhou B , Ji X . Mitochondrial quality control in acute ischemic stroke. J Cereb Blood Flow Metab. 2021;41(12):3157‐3170. 34551609 10.1177/0271678X211046992 PMC8669286

[16] Rutecki PA . Neuronal excitability: voltage‐dependent currents and synaptic transmission. J Clin Neurophysiol. 1992;9(2):195‐211. 1375602

[17] Murphy TH , Li P , Betts K , Liu R . Two‐photon imaging of stroke onset in vivo reveals that NMDA‐receptor independent ischemic depolarization is the major cause of rapid reversible damage to dendrites and spines. J Neurosci. 2008;28(7):1756‐1772. 18272696 10.1523/JNEUROSCI.5128-07.2008 PMC6671530

[18] Choi DW . Excitotoxicity: still hammering the ischemic brain in 2020. Front Neurosci. 2020;14:579953. 33192266 10.3389/fnins.2020.579953 PMC7649323

[19] Shen Z , Xiang M , Chen C , et al. Glutamate excitotoxicity: potential therapeutic target for ischemic stroke. Biomed Pharmacother. 2022;151:113125. 35609367 10.1016/j.biopha.2022.113125

[20] Zhang X , Peng K , Zhang X . The function of the NMDA receptor in hypoxic‐ischemic encephalopathy. Front Neurosci. 2020;14:567665. 33117117 10.3389/fnins.2020.567665 PMC7573650

[21] Achzet LM , Davison CJ , Shea M , Sturgeon I , Jackson DA . Oxidative stress underlies the ischemia/reperfusion‐induced internalization and degradation of AMPA receptors. Int J Mol Sci. 2021;22(2):717. 33450848 10.3390/ijms22020717 PMC7828337

[22] Verma M , Lizama BN , Chu CT . Excitotoxicity, calcium and mitochondria: a triad in synaptic neurodegeneration. Transl Neurodegener. 2022;11(1):3. 35078537 10.1186/s40035-021-00278-7 PMC8788129

[23] Girouard H , Wang G , Gallo EF , et al. NMDA receptor activation increases free radical production through nitric oxide and NOX2. J Neurosci. 2009;29(8):2545‐2552. 19244529 10.1523/JNEUROSCI.0133-09.2009 PMC2669930

[24] Waring P . Redox active calcium ion channels and cell death. Arch Biochem Biophys. 2005;434(1):33‐42. 15629106 10.1016/j.abb.2004.08.001

[25] Gomez‐Castro F , Zappettini S , Pressey JC , et al. Convergence of adenosine and GABA signaling for synapse stabilization during development. Science. 2021;374(6568):eabk2055. 34735259 10.1126/science.abk2055

[26] Schwartz‐Bloom RD , Sah R . Gamma‐aminobutyric acid(A) neurotransmission and cerebral ischemia. J Neurochem. 2001;77(2):353‐371. 11299298 10.1046/j.1471-4159.2001.00274.x

[27] Zhan RZ , Nadler JV , Schwartz‐Bloom RD . Depressed responses to applied and synaptically‐released GABA in CA1 pyramidal cells, but not in CA1 interneurons, after transient forebrain ischemia. J Cereb Blood Flow Metab. 2006;26(1):112‐124. 15959457 10.1038/sj.jcbfm.9600171

[28] Karantali E , Kazis D , Papavasileiou V , et al. Serum BDNF Levels in Acute Stroke: A Systematic Review and Meta‐Analysis. Medicina (Kaunas). 2021;57(3):297. 33809965 10.3390/medicina57030297 PMC8004775

[29] Zhou J , du T , Li B , Rong Y , Verkhratsky A , Peng L . Crosstalk between MAPK/ERK and PI3K/AKT signal pathways during brain ischemia/reperfusion. ASN Neuro. 2015;7(5):1759091415602463. 26442853 10.1177/1759091415602463 PMC4601130

[30] Sun J , Nan G . The mitogen‐activated protein kinase (MAPK) signaling pathway as a discovery target in stroke. J Mol Neurosci. 2016;59(1):90‐98. 26842916 10.1007/s12031-016-0717-8

[31] Chen B , Wang Y . Cofilin rod formation in neurons impairs neuronal structure and function. CNS Neurol Disord Drug Targets. 2015;14(4):554‐560. 25714964 10.2174/1871527314666150225144052

[32] Shu L , Chen B , Chen B , et al. Brain ischemic insult induces cofilin rod formation leading to synaptic dysfunction in neurons. J Cereb Blood Flow Metab. 2019;39(11):2181‐2195. 29932353 10.1177/0271678X18785567 PMC6827117

[33] Lan X , Han X , Li Q , Yang QW , Wang J . Modulators of microglial activation and polarization after intracerebral haemorrhage. Nat Rev Neurol. 2017;13(7):420‐433. 28524175 10.1038/nrneurol.2017.69 PMC5575938

[34] Wake H , Moorhouse AJ , Jinno S , Kohsaka S , Nabekura J . Resting microglia directly monitor the functional state of synapses in vivo and determine the fate of ischemic terminals. J Neurosci. 2009;29(13):3974‐3980. 19339593 10.1523/JNEUROSCI.4363-08.2009 PMC6665392

[35] Nimmerjahn A , Kirchhoff F , Helmchen F . Resting microglial cells are highly dynamic surveillants of brain parenchyma in vivo. Science. 2005;308(5726):1314‐1318. 15831717 10.1126/science.1110647

[36] Tremblay ME , Stevens B , Sierra A , Wake H , Bessis A , Nimmerjahn A . The role of microglia in the healthy brain. J Neurosci. 2011;31(45):16064‐16069. 22072657 10.1523/JNEUROSCI.4158-11.2011 PMC6633221

[37] Lan X , Han X , Liu X , Wang J . Inflammatory responses after intracerebral hemorrhage: from cellular function to therapeutic targets. J Cereb Blood Flow Metab. 2019;39(1):184‐186. 30346222 10.1177/0271678X18805675 PMC6311675

[38] Zhang W , Tian T , Gong SX , et al. Microglia‐associated neuroinflammation is a potential therapeutic target for ischemic stroke. Neural Regen Res. 2021;16(1):6‐11. 32788440 10.4103/1673-5374.286954 PMC7818879

[39] Enzmann G , Mysiorek C , Gorina R , et al. The neurovascular unit as a selective barrier to polymorphonuclear granulocyte (PMN) infiltration into the brain after ischemic injury. Acta Neuropathol. 2013;125(3):395‐412. 23269317 10.1007/s00401-012-1076-3 PMC3578720

[40] Qin C , Zhou LQ , Ma XT , et al. Dual functions of microglia in ischemic stroke. Neurosci Bull. 2019;35(5):921‐933. 31062335 10.1007/s12264-019-00388-3 PMC6754485

[41] Ju F , Ran Y , Zhu L , et al. Increased BBB permeability enhances activation of microglia and exacerbates loss of dendritic spines after transient global cerebral ischemia. Front Cell Neurosci. 2018;12:236. 30123113 10.3389/fncel.2018.00236 PMC6085918

[42] Luo XL , Liu SY , Wang LJ , et al. A tetramethoxychalcone from *Chloranthus henryi* suppresses lipopolysaccharide‐induced inflammatory responses in BV2 microglia. Eur J Pharmacol. 2016;774:135‐143. 26852953 10.1016/j.ejphar.2016.02.013

[43] Alsbrook DL , di Napoli M , Bhatia K , et al. Neuroinflammation in acute ischemic and hemorrhagic stroke. Curr Neurol Neurosci Rep. 2023;23(8):407‐431. 37395873 10.1007/s11910-023-01282-2 PMC10544736

[44] Zhu H , Wang Z , Yu J , et al. Role and mechanisms of cytokines in the secondary brain injury after intracerebral hemorrhage. Prog Neurobiol. 2019;178:101610. 30923023 10.1016/j.pneurobio.2019.03.003

[45] Hong S , Beja‐Glasser VF , Nfonoyim BM , et al. Complement and microglia mediate early synapse loss in Alzheimer mouse models. Science. 2016;352(6286):712‐716. 27033548 10.1126/science.aad8373 PMC5094372

[46] Stevens B , Allen NJ , Vazquez LE , et al. The classical complement cascade mediates CNS synapse elimination. Cell. 2007;131(6):1164‐1178. 18083105 10.1016/j.cell.2007.10.036

[47] Schilling M , Besselmann M , Müller M , Strecker JK , Ringelstein EB , Kiefer R . Predominant phagocytic activity of resident microglia over hematogenous macrophages following transient focal cerebral ischemia: an investigation using green fluorescent protein transgenic bone marrow chimeric mice. Exp Neurol. 2005;196(2):290‐297. 16153641 10.1016/j.expneurol.2005.08.004

[48] Fuhrmann M , Bittner T , Jung CKE , et al. Microglial Cx3cr1 knockout prevents neuron loss in a mouse model of Alzheimer's disease. Nat Neurosci. 2010;13(4):411‐413. 20305648 10.1038/nn.2511 PMC4072212

[49] Neher JJ , Emmrich JV , Fricker M , Mander PK , Théry C , Brown GC . Phagocytosis executes delayed neuronal death after focal brain ischemia. Proc Natl Acad Sci USA. 2013;110(43):E4098‐E4107. 24101459 10.1073/pnas.1308679110 PMC3808587

[50] Chen S , Peng J , Sherchan P , et al. TREM2 activation attenuates neuroinflammation and neuronal apoptosis via PI3K/Akt pathway after intracerebral hemorrhage in mice. J Neuroinflammation. 2020;17(1):168. 32466767 10.1186/s12974-020-01853-x PMC7257134

[51] Qiao HB , Li J , Lv LJ , et al. Eupatilin inhibits microglia activation and attenuates brain injury in intracerebral hemorrhage. Exp Ther Med. 2018;16(5):4005‐4009. 30344678 10.3892/etm.2018.6699 PMC6176204

[52] Shi X , Luo L , Wang J , et al. Stroke subtype‐dependent synapse elimination by reactive gliosis in mice. Nat Commun. 2021;12(1):6943. 34836962 10.1038/s41467-021-27248-x PMC8626497

[53] Alawieh AM , Langley EF , Feng W , Spiotta AM , Tomlinson S . Complement‐dependent synaptic uptake and cognitive decline after stroke and reperfusion therapy. J Neurosci. 2020;40(20):4042‐4058. 32291326 10.1523/JNEUROSCI.2462-19.2020 PMC7219298

[54] Abbott NJ , Ronnback L , Hansson E . Astrocyte‐endothelial interactions at the blood‐brain barrier. Nat Rev Neurosci. 2006;7(1):41‐53. 16371949 10.1038/nrn1824

[55] Halassa MM , Haydon PG . Integrated brain circuits: astrocytic networks modulate neuronal activity and behavior. Annu Rev Physiol. 2010;72:335‐355. 20148679 10.1146/annurev-physiol-021909-135843 PMC3117429

[56] Chung WS , Allen NJ , Eroglu C . Astrocytes control synapse formation, function, and elimination. Cold Spring Harb Perspect Biol. 2015;7(9):a020370. 25663667 10.1101/cshperspect.a020370 PMC4527946

[57] Chung WS , Clarke LE , Wang GX , et al. Astrocytes mediate synapse elimination through MEGF10 and MERTK pathways. Nature. 2013;504(7480):394‐400. 24270812 10.1038/nature12776 PMC3969024

[58] Schafer DP , Lehrman EK , Kautzman AG , et al. Microglia sculpt postnatal neural circuits in an activity and complement‐dependent manner. Neuron. 2012;74(4):691‐705. 22632727 10.1016/j.neuron.2012.03.026 PMC3528177

[59] Paolicelli RC , Bolasco G , Pagani F , et al. Synaptic pruning by microglia is necessary for normal brain development. Science. 2011;333(6048):1456‐1458. 21778362 10.1126/science.1202529

[60] Morizawa YM , Hirayama Y , Ohno N , et al. Reactive astrocytes function as phagocytes after brain ischemia via ABCA1‐mediated pathway. Nat Commun. 2017;8(1):28. 28642575 10.1038/s41467-017-00037-1 PMC5481424

[61] Yamagata K . Astrocyte‐induced synapse formation and ischemic stroke. J Neurosci Res. 2021;99(5):1401‐1413. 33604930 10.1002/jnr.24807

[62] Liauw J , Hoang S , Choi M , et al. Thrombospondins 1 and 2 are necessary for synaptic plasticity and functional recovery after stroke. J Cereb Blood Flow Metab. 2008;28(10):1722‐1732. 18594557 10.1038/jcbfm.2008.65

[63] Zisis E , Keller D , Kanari L , et al. Digital reconstruction of the neuro‐glia‐vascular architecture. Cereb Cortex. 2021;31(12):5686‐5703. 34387659 10.1093/cercor/bhab254 PMC8568010

[64] Li L , Zhou J , Han L , et al. The specific role of reactive astrocytes in stroke. Front Cell Neurosci. 2022;16:850866. 35321205 10.3389/fncel.2022.850866 PMC8934938

[65] Heller JP , Odii T , Zheng K , Rusakov DA . Imaging tripartite synapses using super‐resolution microscopy. Methods. 2020;174:81‐90. 31153907 10.1016/j.ymeth.2019.05.024 PMC7144327

[66] Michiels C , Arnould T , Remacle J . Endothelial cell responses to hypoxia: initiation of a cascade of cellular interactions. Biochim Biophys Acta. 2000;1497(1):1‐10. 10838154 10.1016/s0167-4889(00)00041-0

[67] Han F , Shirasaki Y , Fukunaga K . Microsphere embolism‐induced endothelial nitric oxide synthase expression mediates disruption of the blood‐brain barrier in rat brain. J Neurochem. 2006;99(1):97‐106. 16987238 10.1111/j.1471-4159.2006.04048.x

[68] Ma Y , Jiang L , Wang L , et al. Endothelial progenitor cell transplantation alleviated ischemic brain injury via inhibiting C3/C3aR pathway in mice. J Cereb Blood Flow Metab. 2020;40(12):2374‐2386. 31865842 10.1177/0271678X19892777 PMC7820683

[69] Ma Y , Liu Z , Jiang L , et al. Endothelial progenitor cell transplantation attenuates synaptic loss associated with enhancing complement receptor 3‐dependent microglial/macrophage phagocytosis in ischemic mice. J Cereb Blood Flow Metab. 2023;43(3):379‐392. 36457150 10.1177/0271678X221135841 PMC9941864

[70] Sun J , Yuan Q , Guo L , et al. Brain microvascular endothelial cell‐derived exosomes protect neurons from ischemia‐reperfusion injury in mice. Pharmaceuticals (Basel). 2022;15(10):1287. 36297399 10.3390/ph15101287 PMC9608440

[71] Dalakas MC , Alexopoulos H , Spaeth PJ . Complement in neurological disorders and emerging complement‐targeted therapeutics. Nat Rev Neurol. 2020;16(11):601‐617. 33005040 10.1038/s41582-020-0400-0 PMC7528717

[72] Cornell J , Salinas S , Huang HY , Zhou M . Microglia regulation of synaptic plasticity and learning and memory. Neural Regen Res. 2022;17(4):705‐716. 34472455 10.4103/1673-5374.322423 PMC8530121

[73] Werneburg S , Jung J , Kunjamma RB , et al. Targeted complement inhibition at synapses prevents microglial synaptic engulfment and synapse loss in demyelinating disease. Immunity. 2020;52(1):167‐182.e7. 31883839 10.1016/j.immuni.2019.12.004 PMC6996144

[74] Krukowski K , Chou A , Feng X , et al. Traumatic brain injury in aged mice induces chronic microglia activation, synapse loss, and complement‐dependent memory deficits. Int J Mol Sci. 2018;19(12):3753. 30486287 10.3390/ijms19123753 PMC6321529

[75] Wu T , Dejanovic B , Gandham VD , et al. Complement C3 is activated in human AD brain and is required for neurodegeneration in mouse models of amyloidosis and tauopathy. Cell Rep. 2019;28(8):2111‐2123.e6. 31433986 10.1016/j.celrep.2019.07.060

[76] Chu X , Cao L , Yu Z , et al. Hydrogen‐rich saline promotes microglia M2 polarization and complement‐mediated synapse loss to restore behavioral deficits following hypoxia‐ischemic in neonatal mice via AMPK activation. J Neuroinflammation. 2019;16(1):104. 31103039 10.1186/s12974-019-1488-2 PMC6525972

[77] Pedersen ED , Waje‐Andreassen U , Vedeler CA , Aamodt G , Mollnes TE . Systemic complement activation following human acute ischaemic stroke. Clin Exp Immunol. 2004;137(1):117‐122. 15196251 10.1111/j.1365-2249.2004.02489.x PMC1809093

[78] Pedersen ED , Løberg EM , Vege E , Daha MR , Mæhlen J , Mollnes TE . In situ deposition of complement in human acute brain ischaemia. Scand J Immunol. 2009;69(6):555‐562. 19439017 10.1111/j.1365-3083.2009.02253.x

[79] Szeplaki G , Szegedi R , Hirschberg K , et al. Strong complement activation after acute ischemic stroke is associated with unfavorable outcomes. Atherosclerosis. 2009;204(1):315‐320. 18804761 10.1016/j.atherosclerosis.2008.07.044

[80] Wang C , Yue H , Hu Z , et al. Microglia mediate forgetting via complement‐dependent synaptic elimination. Science. 2020;367(6478):688‐694. 32029629 10.1126/science.aaz2288

[81] Wu M , Chen K , Zhao Y , et al. Normobaric hyperoxia alleviates complement C3‐mediated synaptic pruning and brain injury after intracerebral hemorrhage. CNS Neurosci Ther. 2024;30(3):e14694. 38532579 10.1111/cns.14694 PMC10966135

[82] Wu M , Chen K , Jiang M , et al. High plasma complement C4 levels as a novel predictor of clinical outcome in intracerebral hemorrhage. Front Aging Neurosci. 2023;15:1103278. 36891553 10.3389/fnagi.2023.1103278 PMC9986541

[83] Sellgren CM , Gracias J , Watmuff B , et al. Increased synapse elimination by microglia in schizophrenia patient‐derived models of synaptic pruning. Nat Neurosci. 2019;22(3):374‐385. 30718903 10.1038/s41593-018-0334-7 PMC6410571

[84] Comer AL , Jinadasa T , Sriram B , et al. Increased expression of schizophrenia‐associated gene C4 leads to hypoconnectivity of prefrontal cortex and reduced social interaction. PLoS Biol. 2020;18(1):e3000604. 31935214 10.1371/journal.pbio.3000604 PMC6959572

[85] Yilmaz M , Yalcin E , Presumey J , et al. Overexpression of schizophrenia susceptibility factor human complement C4A promotes excessive synaptic loss and behavioral changes in mice. Nat Neurosci. 2021;24(2):214‐224. 33353966 10.1038/s41593-020-00763-8 PMC8086435

[86] MacDonald JM , Beach MG , Porpiglia E , Sheehan AE , Watts RJ , Freeman MR . The drosophila cell corpse engulfment receptor Draper mediates glial clearance of severed axons. Neuron. 2006;50(6):869‐881. 16772169 10.1016/j.neuron.2006.04.028

[87] Zhou Z , Hartwieg E , Horvitz HR . CED‐1 is a transmembrane receptor that mediates cell corpse engulfment in *C. elegans* . Cell. 2001;104(1):43‐56. 11163239 10.1016/s0092-8674(01)00190-8

[88] Hamon Y , Trompier D , Ma Z , et al. Cooperation between engulfment receptors: the case of ABCA1 and MEGF10. PLoS One. 2006;1(1):e120. 17205124 10.1371/journal.pone.0000120 PMC1762421

[89] Wu HH , Bellmunt E , Scheib JL , et al. Glial precursors clear sensory neuron corpses during development via Jedi‐1, an engulfment receptor. Nat Neurosci. 2009;12(12):1534‐1541. 19915564 10.1038/nn.2446 PMC2834222

[90] Kinchen JM , Cabello J , Klingele D , et al. Two pathways converge at CED‐10 to mediate Actin rearrangement and corpse removal in *C. elegans* . Nature. 2005;434(7029):93‐99. 15744306 10.1038/nature03263

[91] Prasad D , Rothlin CV , Burrola P , et al. TAM receptor function in the retinal pigment epithelium. Mol Cell Neurosci. 2006;33(1):96‐108. 16901715 10.1016/j.mcn.2006.06.011

[92] Duncan JL , LaVail MM , Yasumura D , et al. An RCS‐like retinal dystrophy phenotype in mer knockout mice. Invest Ophthalmol Vis Sci. 2003;44(2):826‐838. 12556419 10.1167/iovs.02-0438

[93] Finnemann SC . Focal adhesion kinase signaling promotes phagocytosis of integrin‐bound photoreceptors. EMBO J. 2003;22(16):4143‐4154. 12912913 10.1093/emboj/cdg416 PMC175805

[94] Wu Y , Singh S , Georgescu MM , Birge RB . A role for Mer tyrosine kinase in alphavbeta5 integrin‐mediated phagocytosis of apoptotic cells. J Cell Sci. 2005;118(Pt 3):539‐553. 15673687 10.1242/jcs.01632

[95] Chicheportiche Y , Bourdon PR , Xu H , et al. TWEAK, a new secreted ligand in the tumor necrosis factor family that weakly induces apoptosis. J Biol Chem. 1997;272(51):32401‐32410. 9405449 10.1074/jbc.272.51.32401

[96] Winkles JA . The TWEAK‐Fn14 cytokine‐receptor axis: discovery, biology and therapeutic targeting. Nat Rev Drug Discov. 2008;7(5):411‐425. 18404150 10.1038/nrd2488 PMC3018765

[97] Burkly LC , Michaelson JS , Zheng TS . TWEAK/Fn14 pathway: an immunological switch for shaping tissue responses. Immunol Rev. 2011;244(1):99‐114. 22017434 10.1111/j.1600-065X.2011.01054.x

[98] Cheadle L , Tzeng CP , Kalish BT , et al. Visual experience‐dependent expression of Fn14 is required for retinogeniculate refinement. Neuron. 2018;99(3):525‐539.e10. 30033152 10.1016/j.neuron.2018.06.036 PMC6101651

[99] Cheadle L , Rivera SA , Phelps JS , et al. Sensory experience engages microglia to shape neural connectivity through a non‐phagocytic mechanism. Neuron. 2020;108(3):451‐468.e9. 32931754 10.1016/j.neuron.2020.08.002 PMC7666095

[100] Potrovita I , Zhang W , Burkly L , et al. Tumor necrosis factor‐like weak inducer of apoptosis‐induced neurodegeneration. J Neurosci. 2004;24(38):8237‐8244. 15385607 10.1523/JNEUROSCI.1089-04.2004 PMC6729692

[101] Yepes M , Brown SAN , Moore EG , Smith EP , Lawrence DA , Winkles JA . A soluble Fn14‐fc decoy receptor reduces infarct volume in a murine model of cerebral ischemia. Am J Pathol. 2005;166(2):511‐520. 15681834 10.1016/S0002-9440(10)62273-0 PMC1602337

[102] Inta I , Frauenknecht K , Dörr H , et al. Induction of the cytokine TWEAK and its receptor Fn14 in ischemic stroke. J Neurol Sci. 2008;275(1–2):117‐120. 18793781 10.1016/j.jns.2008.08.005

[103] Nagy D , Ennis KA , Wei R , et al. Developmental synaptic regulator, TWEAK/Fn14 signaling, is a determinant of synaptic function in models of stroke and neurodegeneration. Proc Natl Acad Sci USA. 2021;118(6):e2001679118. 33526652 10.1073/pnas.2001679118 PMC8017933

[104] Sun H , He X , Tao X , et al. The CD200/CD200R signaling pathway contributes to spontaneous functional recovery by enhancing synaptic plasticity after stroke. J Neuroinflammation. 2020;17(1):171. 32473633 10.1186/s12974-020-01845-x PMC7260848

[105] Feng D , Huang A , Yan W , Chen D . CD200 dysfunction in neuron contributes to synaptic deficits and cognitive impairment. Biochem Biophys Res Commun. 2019;516(4):1053‐1059. 31277944 10.1016/j.bbrc.2019.06.134

[106] Cox FF , Carney D , Miller AM , Lynch MA . CD200 fusion protein decreases microglial activation in the hippocampus of aged rats. Brain Behav Immun. 2012;26(5):789‐796. 22041297 10.1016/j.bbi.2011.10.004

[107] Costello DA , Lyons A , Denieffe S , Browne TC , Cox FF , Lynch MA . Long term potentiation is impaired in membrane glycoprotein CD200‐deficient mice: a role for toll‐like receptor activation. J Biol Chem. 2011;286(40):34722‐34732. 21835925 10.1074/jbc.M111.280826 PMC3186410

[108] Sharma N , Classen J , Cohen LG . Neural plasticity and its contribution to functional recovery. Handb Clin Neurol. 2013;110:3‐12. 23312626 10.1016/B978-0-444-52901-5.00001-0 PMC4880010

[109] Sun H , Li A , Hou T , et al. Neurogenesis promoted by the CD200/CD200R signaling pathway following treadmill exercise enhances post‐stroke functional recovery in rats. Brain Behav Immun. 2019;82:354‐371. 31513876 10.1016/j.bbi.2019.09.005

[110] Wang S , Wang C , Wang L , Cai Z . Minocycline inhibits mTOR signaling activation and alleviates behavioral deficits in the Wistar rats with acute ischemia stroke. CNS Neurol Disord Drug Targets. 2020;19(10):791‐799. 32867663 10.2174/1871527319999200831153748

[111] Liu H , Zhang Z , Zang C , et al. GJ‐4 ameliorates memory impairment in focal cerebral ischemia/reperfusion of rats via inhibiting JAK2/STAT1‐mediated neuroinflammation. J Ethnopharmacol. 2021;267:113491. 33091490 10.1016/j.jep.2020.113491

[112] Cai L , Li W , Lu X , et al. Neural Fuyuan formula promotes neural plasticity through BDNF/Trkbeta signaling pathway. Ann Palliat Med. 2021;10(3):2926‐2934. 33691458 10.21037/apm-19-533

[113] Liang X , Shi L , Wang M , et al. Folic acid ameliorates synaptic impairment following cerebral ischemia/reperfusion injury via inhibiting excessive activation of NMDA receptors. J Nutr Biochem. 2023;112:109209. 36370927 10.1016/j.jnutbio.2022.109209

[114] Shi YJ , Sun LL , Ji X , Shi R , Xu F , Gu JH . Neuroprotective effects of oleanolic acid against cerebral ischemia‐reperfusion injury in mice. Exp Neurol. 2021;343:113785. 34153323 10.1016/j.expneurol.2021.113785

[115] Zhang Z , Cao X , Bao X , Zhang Y , Xu Y , Sha D . Cocaine‐ and amphetamine‐regulated transcript protects synaptic structures in neurons after ischemic cerebral injury. Neuropeptides. 2020;81:102023. 32005500 10.1016/j.npep.2020.102023

[116] Xie L , Xie Y , Mao G , et al. Decreased spasticity of Baishaoluoshi decoction through the BDNF/TrKB‐KCC2 pathway on poststroke spasticity rats. Neuroreport. 2021;32(14):1183‐1191. 34284448 10.1097/WNR.0000000000001709 PMC8389354

[117] Fu XQ , Lan R , Zhang Y , Wang MM , Zou XH , Wang WW . Effect of Xiaoxuming decoction on synaptic plasticity following acute cerebral ischemia‐reperfusion in rats. Zhongguo Zhong Yao Za Zhi. 2023;48(14):3882‐3889. 37475080 10.19540/j.cnki.cjcmm.20230403.403

[118] Ou Z , Zhao M , Xu Y , et al. Huangqi Guizhi Wuwu decoction promotes M2 microglia polarization and synaptic plasticity via Sirt1/NF‐kappaB/NLRP3 pathway in MCAO rats. Aging (Albany NY). 2023;15(19):10031‐10056. 37650573 10.18632/aging.204989 PMC10599726

[119] Sadigh‐Eteghad S , Geranmayeh MH , Majdi A , Salehpour F , Mahmoudi J , Farhoudi M . Intranasal cerebrolysin improves cognitive function and structural synaptic plasticity in photothrombotic mouse model of medial prefrontal cortex ischemia. Neuropeptides. 2018;71:61‐69. 30054019 10.1016/j.npep.2018.07.002

[120] Gao X , Li R , Luo L , Liao C , Yang H , Mao S . Alpha‐asarone ameliorates neurological dysfunction of subarachnoid hemorrhagic rats in both acute and recovery phases via regulating the CaMKII‐dependent pathways. Transl Stroke Res. 2023;15:476‐494. 36781743 10.1007/s12975-023-01139-3

[121] Huo Y , Li J , Qin X , et al. Efficacy of folic acid therapy in primary prevention of stroke among adults with hypertension in China: the CSPPT randomized clinical trial. JAMA. 2015;313(13):1325‐1335. 25771069 10.1001/jama.2015.2274

[122] Han YW , Liu XJ , Zhao Y , Li XM . Role of Oleanolic acid in maintaining BBB integrity by targeting p38MAPK/VEGF/Src signaling pathway in rat model of subarachnoid hemorrhage. Eur J Pharmacol. 2018;839:12‐20. 30240794 10.1016/j.ejphar.2018.09.018

[123] Lin K , Zhang Z , Zhang Z , et al. Oleanolic acid alleviates cerebral ischemia/reperfusion injury via regulation of the GSK‐3beta/HO‐1 signaling pathway. Pharmaceuticals (Basel). 2021;15(1):1. 35056059 10.3390/ph15010001 PMC8781522

[124] Cole GJ , Halfter W . Agrin: an extracellular matrix heparan sulfate proteoglycan involved in cell interactions and synaptogenesis. Perspect Dev Neurobiol. 1996;3(4):359‐371. 9117266

[125] Zhang P , Yang L , Li G , et al. Agrin involvement in synaptogenesis induced by exercise in a rat model of experimental stroke. Neurorehabil Neural Repair. 2020;34(12):1124‐1137. 33135566 10.1177/1545968320969939

[126] Wang Y , Qiu B , Liu J , Zhu WG , Zhu S . Cocaine‐ and amphetamine‐regulated transcript facilitates the neurite outgrowth in cortical neurons after oxygen and glucose deprivation through PTN‐dependent pathway. Neuroscience. 2014;277:103‐110. 25010400 10.1016/j.neuroscience.2014.06.064

[127] Ko G , Kim J , Jeon YJ , Lee D , Baek HM , Chang KA . Salvia miltiorrhiza alleviates memory deficit induced by ischemic brain injury in a transient MCAO mouse model by inhibiting ferroptosis. Antioxidants (Basel). 2023;12(4):785. 37107160 10.3390/antiox12040785 PMC10135292

[128] Wang L , Wang Y , Chen YJ , et al. Rhynchophylline ameliorates cerebral ischemia by improving the synaptic plasticity in a middle cerebral artery occlusion induced stroke model. Eur J Pharmacol. 2023;940:175390. 36400162 10.1016/j.ejphar.2022.175390

[129] Chi X , Wang L , Liu H , Zhang Y , Shen W . Post‐stroke cognitive impairment and synaptic plasticity: a review about the mechanisms and Chinese herbal drugs strategies. Front Neurosci. 2023;17:1123817. 36937659 10.3389/fnins.2023.1123817 PMC10014821

[130] Liu Y , Wang S , Kan J , et al. Chinese herbal medicine interventions in neurological disorder therapeutics by regulating glutamate signaling. Curr Neuropharmacol. 2020;18(4):260‐276. 31686629 10.2174/1570159X17666191101125530 PMC7327939

[131] Qin S , Zhang Z , Zhao Y , et al. The impact of acupuncture on neuroplasticity after ischemic stroke: a literature review and perspectives. Front Cell Neurosci. 2022;16:817732. 36439200 10.3389/fncel.2022.817732 PMC9685811

[132] Xing Y , Zhang Y , Li C , et al. Repetitive transcranial magnetic stimulation of the brain after ischemic stroke: mechanisms from animal models. Cell Mol Neurobiol. 2023;43(4):1487‐1497. 35917043 10.1007/s10571-022-01264-x PMC11412424

[133] Vink J , van Lieshout ECC , Otte WM , et al. Continuous theta‐burst stimulation of the Contralesional primary motor cortex for promotion of upper limb recovery after stroke: a randomized controlled trial. Stroke. 2023;54(8):1962‐1971. 37345546 10.1161/STROKEAHA.123.042924 PMC10358447

[134] Feng W , Plow EB , Paik NJ . Transcranial magnetic stimulation for Poststroke motor recovery: what we have learned. Stroke. 2023;54(8):1972‐1973. 37345547 10.1161/STROKEAHA.123.043536

[135] Li C , Ke C , Su Y , Wan C . Exercise intervention promotes the growth of synapses and regulates neuroplasticity in rats with ischemic stroke through exosomes. Front Neurol. 2021;12:752595. 34777222 10.3389/fneur.2021.752595 PMC8581302

[136] Li C , Hu J , Liu W , et al. Exercise intervention modulates synaptic plasticity by inhibiting excessive microglial activation via exosomes. Front Cell Neurosci. 2022;16:953640. 35928570 10.3389/fncel.2022.953640 PMC9345504

[137] Chen Z , Gao M , Su Y , Liu P , Sun B . Running promotes transformation of brain astrocytes into neuroprotective reactive astrocytes and synaptic formation by targeting Gpc6 through the STAT3 pathway. Front Physiol. 2021;12:633618. 34122124 10.3389/fphys.2021.633618 PMC8189178

